# A rare case of MRI of myxoid hepatocellular adenoma with gadoxetic acid uptake

**DOI:** 10.1259/bjrcr.20230033

**Published:** 2023-10-18

**Authors:** Tze Wei Wilson Yang, Naveen Mayavel, Jan Frank Gerstenmaier, Ahmad Aga, Charles Pilgrim, Helen Kavnoudias

**Affiliations:** 1 Department of General Surgery, Hepato-Pancreatico-Biliary Unit, Alfred Hospital, Melbourne, Australia; 2 Department of Radiology, Alfred Hospital, Melbourne, Australia; 3 Department of Anatomical Pathology, Cabrini Pathology, Malvern, Australia; 4 Department of Surgery, Monash University, Melbourne, Australia

## Abstract

This is the first case report of 43-year-old lady with a myxoid hepatic adenoma which demonstrated significant contrast uptake during hepatobiliary phase imaging. This highlights the potential for a missed diagnosis and likely subsequent malignant transformation in a young patient in whom it was initially presumed to be focal nodular hyperplasia with no further surveillance.

## Clinical presentation

A 43-year-old female had a palpable incidental liver mass identified during an abdominal examination at the time of consultation for pelvic floor weakness. She did not have any gastrointestinal symptoms, changes in bowel habits, nor systemic features of malignancy or infection. She reported excessive alcohol consumption, drinking a bottle of wine every night for the last 10 years, but displayed no evidence of liver cirrhosis. She had no significant family history of liver disease or malignancy.

## Investigations and imaging findings

Laboratory investigations revealed normal full blood examination panel, with normal platelet count of 224 × 10^9  l^−1^, international normalised ratio of 0.9 and plasma ammonia of 32 μmol l^−1^. Liver function tests were unremarkable including an albumin of 50 g l^−1^, bilirubin of 17 μmol l^−1^, normal transaminases, alkaline phosphatase of 63 U l^−1^ and gamma-glutamyl transferase of 32 U l^−1^. Tumour markers including carcinoembryonic antigen, cancer antigen 15–3, cancer antigen 125, cancer antigen 19–9 were within normal limits. Hepatitic screen for hepatitis B surface antigen and hepatitis C antibodies were negative.

**Figure 1.**
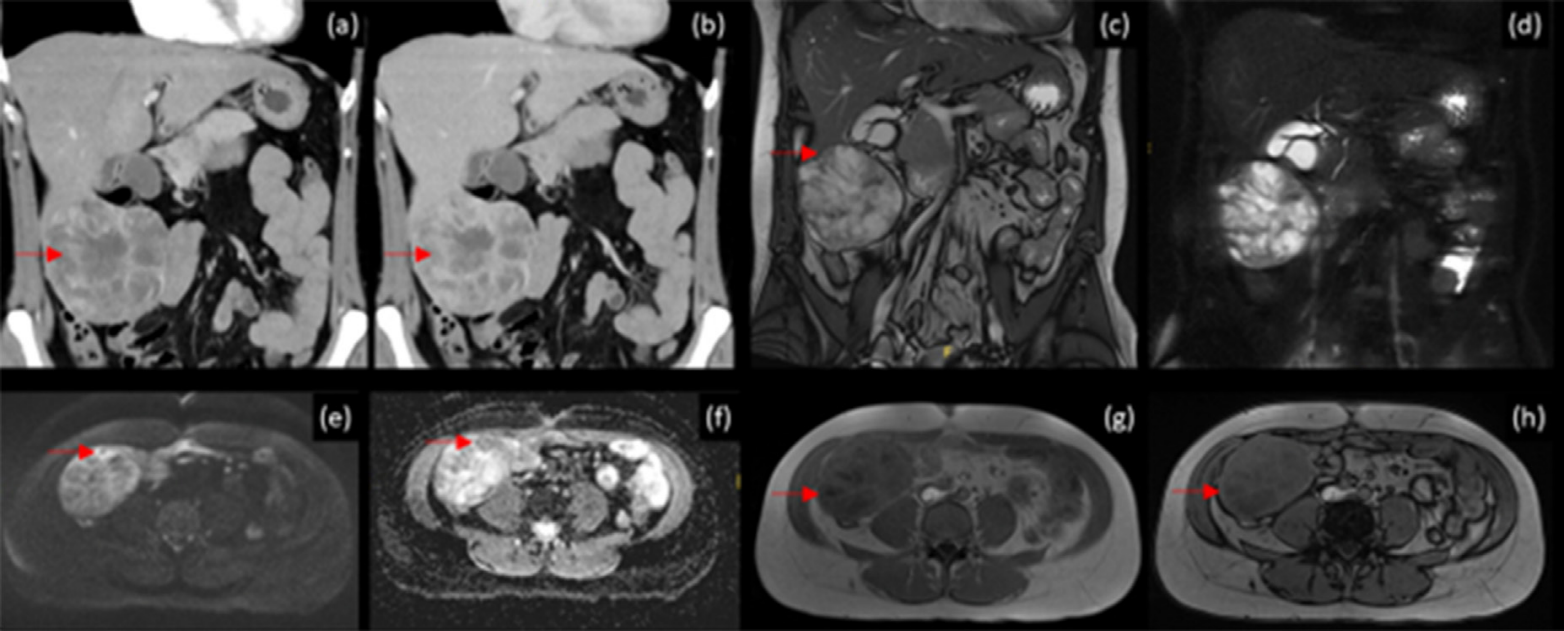
(**a**) Coronal CT with intravenous contrast in arterial phase demonstrates the exophytic mass at the inferior liver edge with lace-like arterial hyperenhancement. (**b**) Coronal CT with intravenous contrast in portal venous phase demonstrates progressive nodular enhancement (arrows in (**a**) and (**b**)). (**c**) Coronal *T*_2_ weighted MRI of the liver demonstrates demarcation of the mass from the liver (arrow). (**d**) Coronal *T*_2_ weighted fat suppressed MRI demonstrates internal water signal structures. (**e**) Diffusion-weighted imaging with *b* = 1000 demonstrating areas of high signal with corresponding low values on apparent diffusion coefficient map (**f**) indicated by arrows. (**g**) *T*_1_ weighted in-phase and (**h**) *T*_1_ weighted opposed-phase imaging with no indication of fractional intravoxel fat but areas with slightly increased signal on T1-opposed phase (arrows) without definite corresponding low T2 signal.

CT scan of the liver demonstrated a segment 5/6 large exophytic solid mass with multiple central hypodensities on the unenhanced imaging. On arterial phase, there was hypervascular curvilinear and nodular internal enhancement with hypoenhancing hypodense components. On the portal venous phase, there was some degree of gradual enhancement of the hypodense areas (Figure 1), which demonstrated gradual enhancement on the subsequent delayed imaging. On the 10 min delayed phase, the solid component of the mass was hyperenhancing compared to the liver parenchyma. The intrahepatic biliary ducts were non-dilated, and portal vein, inferior vena cava and hepatic arteries opacified well (Figure 1). The imaging differential diagnosis at this point included atypical focal nodular hyperplasia (FNH) and haemangioma.

**Figure 2.**
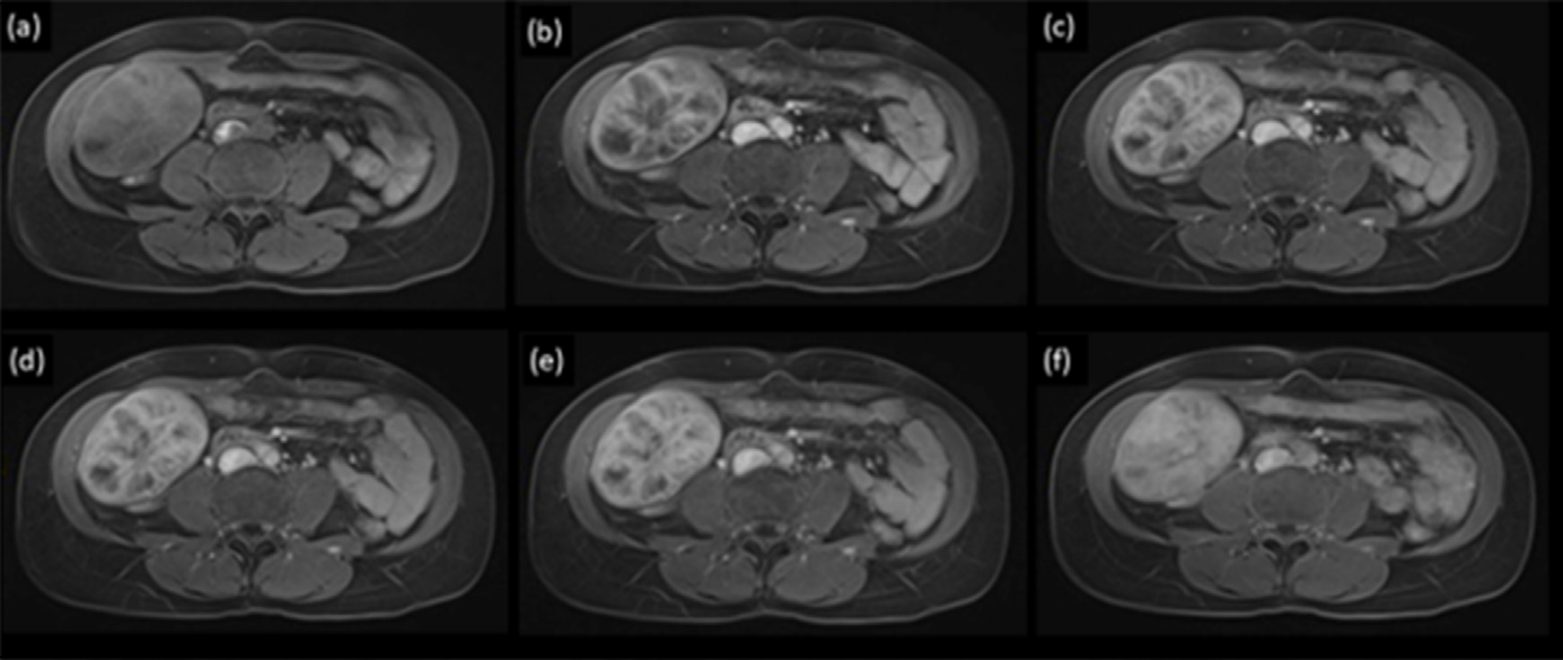
Dynamic *T*_1_ weighted fat-suppressed axial MRI pre- (**a**) and post-intravenous Gd-EOB (Primovist^®^) contrast in arterial (**b**), 30 s (**c**), 60 s (**d**), 90 s (**e**) and 20 min delayed (**f**) phases demonstrating enhancement and retention of hepatocyte specific contrast agent within the mass.

**Figure 3.**
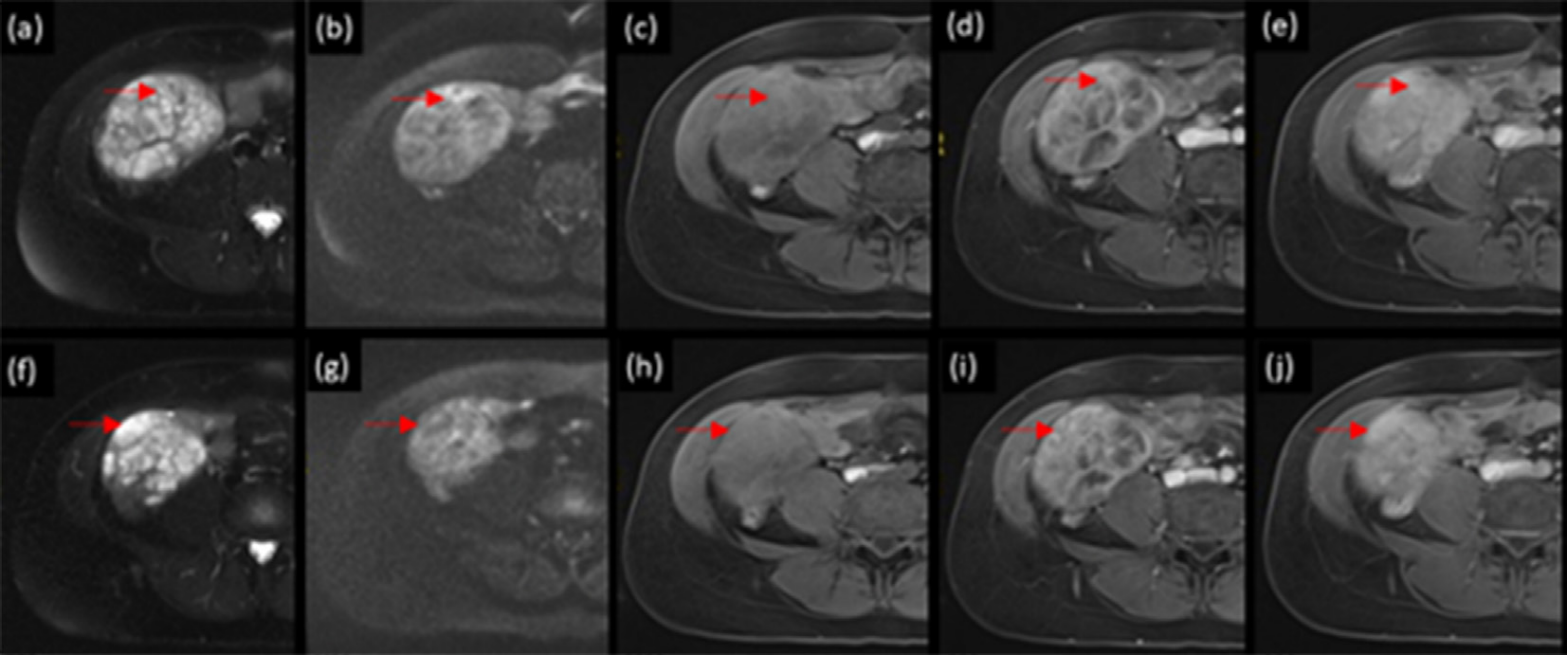
More detailed examination of areas of lower (**a**) and higher (**f**) T2-signal in the mass. Diffusion-weighted imaging, unenhanced, arterial phase and hepatobiliary phase post intravenous Gd-EOB (Primovist^®^) *T*_1_ weighted fat-suppressed sequences corresponding to lower T2 signal areas (**b–e**) and higher T2 signal areas (**g–j**). Uptake of Gd-EOB (Primovist^®^) is present corresponding to both lower (**e**) and higher (**j**) T2 signal areas

Therefore, MRI of the liver with gadoxetic acid (Gd-EOB) (Primovist^®^, Bayer-Schering, Berlin, Germany) was performed. At MRI, a large heterogeneous mass (9.4 × 6.1 × 8.4 cm) with multiple internal septations which demonstrated intermediate T1 and low T2 signal was evident in S5–S6. There were areas of increased diffusion restriction corresponding to low T2 signal structures. There were also internal circumscribed T2 hyperintense structures with elevated apparent diffusion coefficient value. There was no evidence of lipid or fat content, however, on *T*_1_ weighted opposed-phase sequence, there were areas demonstrating a mild signal increase compared to low signal on *T*_1_ weighted in-phase sequence. This can be seen in haemosiderin deposition due to prior haemorrhages. On dynamic sequences, there was moderate peripheral enhancement in the arterial phase, with minimal centripetal filling in the portal venous phase and no washout. At the 20 min delayed hepatobiliary phase (HBP), there was uptake of Gd-EOB throughout the mass (Figure 2). Both areas of intermediate T2 signal and high T2 signal, the former demonstrating arterial enhancement, showed uptake of Gd-EOB (Figure 3).

## Differential diagnosis

The patient was discussed at a Hepatobiliary Unit’s multidisciplinary meeting with the consensus agreeing that it was indeterminate, but FNH remained in the differential diagnosis based on the mass lesion appearing to retain contrast on HBP. A radioisotope-labelled sulphur colloid scan was suggested in order to demonstrate functioning Kupffer cells which are present in FNH. However, this was not eventually performed as the patient chose the option of lesion resection given ongoing concern about the nature of the pathology.

## Treatment and outcome

**Figure 4.**
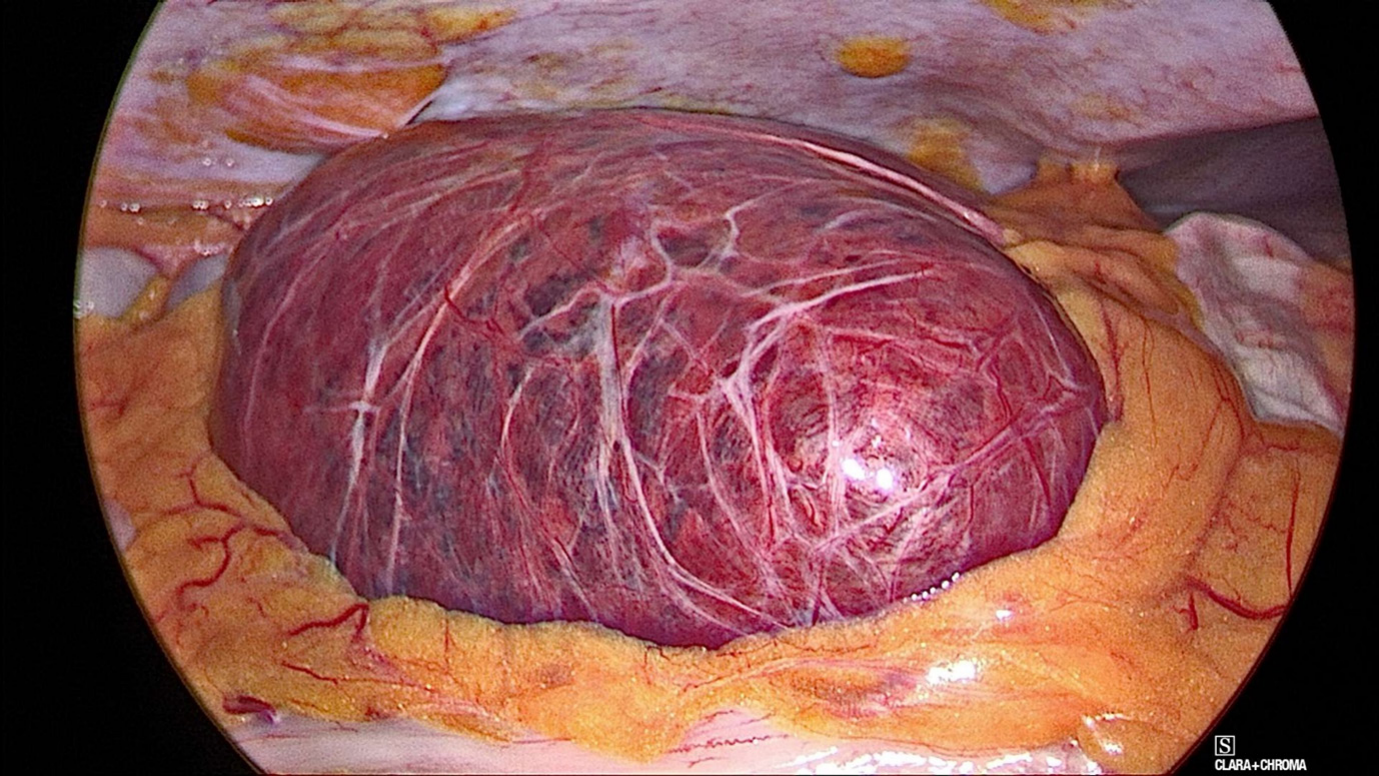
Intraoperative photograph of the segment five liver lesion.

**Figure 5.**
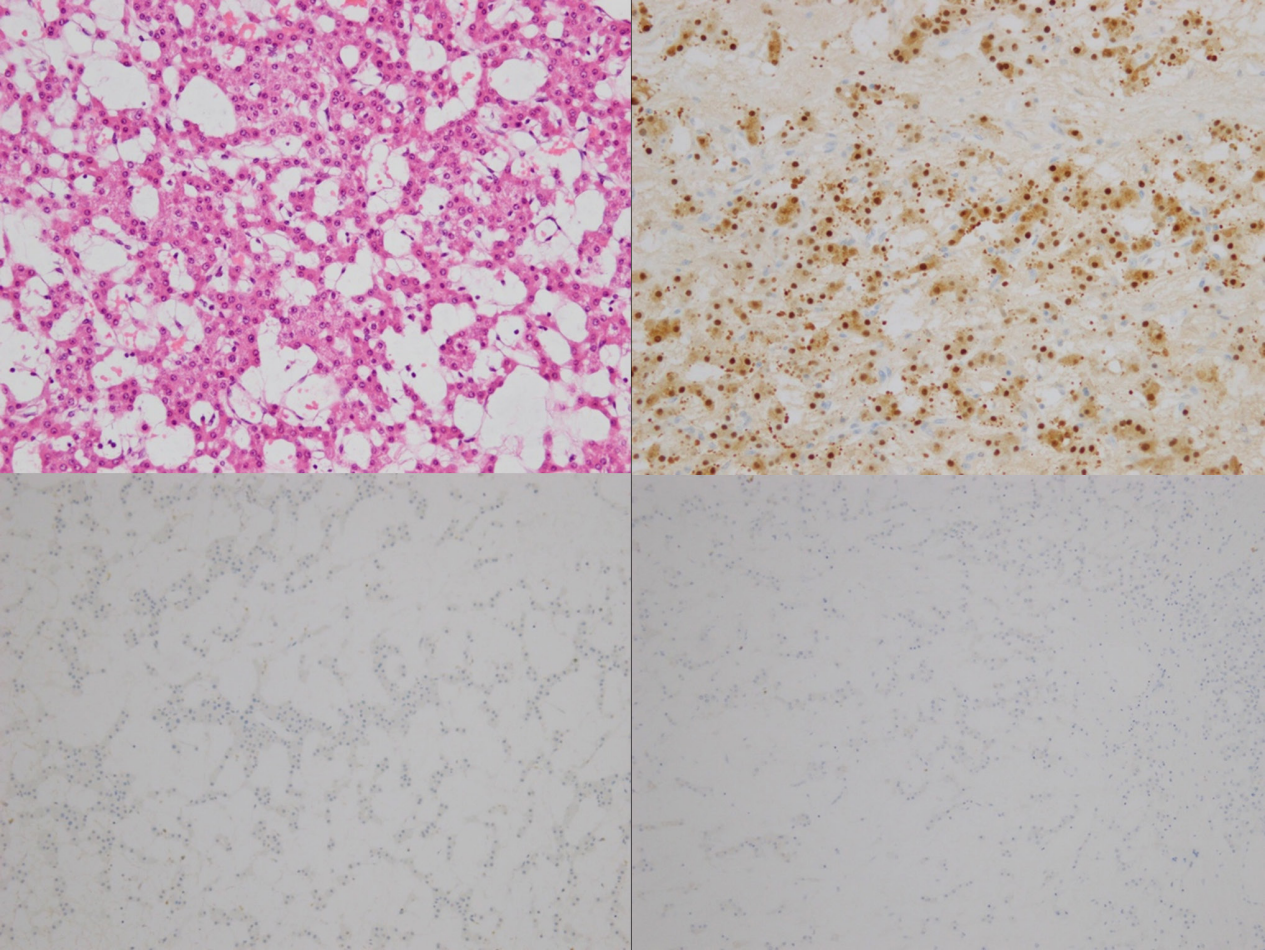
(Top left) Predominantly cords and small nests of bland hepatocytes, embedded within myxoid stroma. Mild thickening of the hepatic plates with intact reticulin meshwork. Scattered arterioles are seen within the lesion with a central area of haemorrhage associated with haemosiderin pigment deposits. Haematoxylin and Eosin x 200. (Top right) Positive immunohistochemistry for Arginase-1. (Bottom left) Negative immunohistochemistry for glutamine synthetase. (Bottom right) Negative immunohistochemistry for glypican 3.

Hence, a segmentectomy of S5 was performed (Figure 4). The resected specimen comprised predominantly cords and small nests of bland hepatocytes, embedded within myxoid stroma microscopically. There was mild thickening of the hepatic plates but the reticulin meshwork was intact. There were areas of haemorrhage associated with haemosiderin pigment deposits. The lesion was positive for arginase, and negative for CRP, glutamine synthetase and glypican 3 (Figure 5). This was conclusive of liver segment five myxoid hepatocellular adenoma (HCA) which was clear of resection margin. The patient had an uncomplicated post-operative course and was discharged home.

## Discussion

To our knowledge, this is the first case report of a myxoid hepatic adenoma which demonstrated significant contrast uptake during HBP imaging, which is normally indicative of functional hepatocytes and an at least rudimentary biliary architecture.^1^

Myxoid hepatic neoplasms (MHN) are a rare variant of hepatic neoplasms^2^ with distinctive disposition of extracellular myxoid material between the hepatic plates^3^ and are characterised by loss of LFANP expression or HNF1A mutation.^4^ There are very few MHN cases reported in the literature to date, but they tend to occur in older-aged persons and carry a high risk of malignant transformation.^3,4^

The previously described MRI appearance of MHN includes markedly increased T2 signal with internal septations and a heterogeneous, progressive, largely centripetal enhancement pattern.^5^ They are described to lack Gd-EOB uptake at HBP imaging.^4,5^ Typically on MRI, FNH are iso- or hypointense on *T*_1_ weighted images, hyper- or isointense on *T*_2_ weighted images, and have a hyperintense central scar which enhances in the delayed phases of contrast-enhanced imaging.^6^ The scar enhancement is typically visible using extracellular, aspecific gadolinium chelate contrast agents and less evident when using Gd-EOB due to the peculiarity of gadolinium chelate which is lacing an interstitial distribution with fast biliary and renal excretion. Gd-EOB is actively transported into functioning hepatocytes via the organic anion transporting polypeptide 1B3 (OATP1B3). HBP enhancement is seen in masses with functioning hepatocytes like FNH, and it is this feature that is used to differentiate it from HCA.^7^ However, it is recognised that a minority of HCA demonstrate Gd-EOB uptake to varying degrees at HBP imaging^8^ and it has been suggested that the degree of the uptake can be used for HCA subtyping,^9,10^ although there is a paucity of literature to address these features. Variable degrees of Gd-EOB uptake can be seen in β-catenin-mutated HCAs, thought to be due to the relationship between the activation of the β-catenin pathway and OATP1B3 expression.^10,11^ Previous case reports on MHN specifically describe no uptake of Gd-EOB. In this case, both areas of high T2 signal within the mass, presumably reflecting myoxid material, as well as intermediate T2-signal structures at the periphery demonstrated uptake in the hepatobiliary phase. Therefore, ‘pooling’ into the myxoid components (no hepatocytes within) may be present, but solid components also demonstrated uptake of hepatobiliary contrast. Mutational analysis was not performed in this case to search for β-catenin mutation.

## Learning points

- This case report aims to highlight the unusual uptake of Gd-EOB on HBP in a patient with myxoid hepatic adenoma.
- This highlights the potential for a missed diagnosis and likely subsequent malignant transformation in a young patient in whom it was initially presumed to be FNH with no further surveillance.
- The differentiation between benign and malignant MHN may not be possible at imaging.
- This case report will further expand the clinical and radiological findings of MHN in the literature.
